# Design and TCAD Simulation of GaN P-i-N Diode with Multi-Drift-Layer and Field-Plate Termination Structures

**DOI:** 10.3390/mi16080839

**Published:** 2025-07-22

**Authors:** Zhibo Yang, Guanyu Wang, Yifei Wang, Pandi Mao, Bo Ye

**Affiliations:** 1 School of Integrated Circuits, Chongqing University of Posts and Telecommunications, Chongqing 400065, China; 2022210967@stu.cqupt.edu.cn; 2 School of Electronic Science and Engineering, Chongqing University of Posts and Telecommunications, Chongqing 400065, China; 2022210826@stu.cqupt.edu.cn; 3 School of Electrical Engineering and Intelligent Manufacturing, Chongqing Metropolitan College of Science and Technology, Chongqing 402167, China; 13181719389@163.com

**Keywords:** gallium nitride, P-i-N diode, edge termination technology, multi-drift layer

## Abstract

Vertical GaN P-i-N diodes exhibit excellent high-voltage performance, fast switching speed, and low conduction losses, making them highly attractive for power applications. However, their breakdown voltage is severely constrained by electric field crowding at device edges. Using silvaco tcad (2019) tools, this work systematically evaluates multiple edge termination techniques, including deep-etched mesa, beveled mesa, and field-plate configurations with both vertical and inclined mesa structures. We present an optimized multi-drift-layer GaN P-i-N diode incorporating field-plate termination and analyze its electrical performance in detail. This study covers forward conduction characteristics including on-state voltage, on-resistance, and their temperature dependence, reverse breakdown behavior examining voltage capability and electric field distribution under different temperatures, and switching performance addressing both forward recovery phenomena, i.e., voltage overshoot and carrier injection dynamics, and reverse recovery characteristics including peak current and recovery time. The comprehensive analysis offers practical design guidelines for developing high-performance GaN power devices.

## 1. Introduction

Gallium nitride (GaN) has garnered increasing attention owing to its superior material properties, such as high electron mobility, high electron saturation velocity, high thermal conductivity, and critical electric field [1,2,3,4]. These excellent material properties enable high breakdown voltage, low on-resistance, and reduced power loss. Vertical GaN power devices have great advantages. By increasing the drift-layer thickness without increasing the device size, higher breakdown voltage can be achieved. They also feature high current capacity, superior heat dissipation, compact chip area, and low dislocation density, making them widely applicable in power electronics [5,6,7]. Vertical GaN P-i-N diodes with high breakdown voltage, low on-resistance, and low reverse leakage current have been extensively studied and made great progress [8,9,10]. However, destructive breakdown and premature avalanche breakdown are induced by the electric field crowding effect at the device edge, PN junction edge, depletion region edge, and electrode edge. In order to alleviate the electric field at the edge of the device and improve the avalanche breakdown ability to achieve high breakdown voltage power devices, edge termination technology has been widely used in power devices to reduce the tip electric field by controlling the depletion region of the junction. These technologies mainly include field plates, ion implantation and plasma treatment, mesa etching, guard rings, junction termination extension, etc. [11,12,13,14]. Notably, these edge termination technologies do not degrade the forward characteristics of devices, and these terminal technologies can also be combined to achieve better device performance.

To approximate the breakdown voltage of an ideal parallel-plane device, in this work, we used TCAD simulation tool to study two mesa-based edge terminal technologies, namely, deep etching mesa and inclined mesa terminal structure, hoping to find a terminal protection structure suitable for vertical GaN P-i-N diodes, and further improve the breakdown characteristics of the device. In addition, considering that the field-plate terminal structure can expand the depletion region along the device interior by sharing the anode reverse bias voltage, so as to improve the breakdown voltage, the field-plate structures of vertical and inclined mesas are also simulated and analyzed in this work.

Building upon the existing mesa termination technology, this work presents an optimized GaN P-i-N diode design featuring a multi-drift-layer structure with integrated field-plate termination [15]. The full mesa field-plate configuration effectively suppresses electric field crowding at device edges, significantly improving breakdown voltage while reducing reverse leakage current. The multi-drift-layer architecture further enhances device performance through carefully engineered doping profiles that optimize both voltage-blocking capability and forward conduction characteristics. Through systematic TCAD simulations, we comprehensively evaluate the electrical performance of this advanced design. The analysis demonstrates excellent forward conduction properties with low on-resistance, superior reverse blocking capability, and robust switching characteristics. The device exhibits particularly stable performance across temperature variations, maintaining reliable operation under high-voltage conditions while showing minimal switching losses. These results validate the effectiveness of the combined multi-drift-layer and field-plate approach for high-performance GaN power devices.

## 2. Structure Design of Different Mesa Terminals of GaN-Based P-i-N Diodes

**Figure 1:**
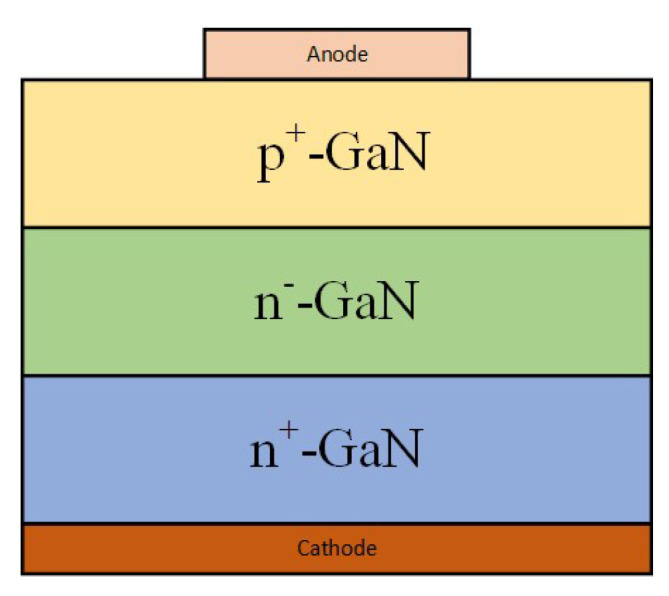
Ideal parallel-plane vertical GaN P-i-N diode structure.

Figure 1 presents the schematic of an ideal parallel-plane GaN P-i-N diode structure, comprising a 500 nm thick p^+^-GaN layer with a doping concentration of 1 × 10^19^ cm^−3^, a 10 µm thick n^-^-GaN drift layer doped at 2 × 10^16^ cm^−3^, and a 2 µm thick n^+^-GaN layer with 2 × 10^18^ cm^−3^ doping concentration. Due to the narrow space charge region in the heavily doped p^+^-GaN layer, the device essentially behaves as a one-sided abrupt junction. The breakdown voltage exhibits negligible dependence on the p^+^-GaN layer thickness, which is intentionally kept thin in both actual devices and our simulation structure to reflect practical designs.

The numerical simulations incorporate key GaN material parameters as listed in Table 1, along with essential physical models including bandgap narrowing, Farahmand-modified Caughey–Thomas mobility model, nitride high-field saturation mobility model, and Fermi–Dirac statistics. These models facilitate the accurate characterization of the device’s electrical behavior across diverse operating conditions.

This study employs TCAD simulations to analyze the electric field distribution and breakdown voltage characteristics under varying reverse bias conditions. The investigation focuses on evaluating how different edge termination structures affect device breakdown performance. For clarity of analysis, we implement selected termination methods in simplified device structures, enabling the precise characterization of their impact on breakdown behavior. These findings will guide the optimization of edge termination designs for GaN P-i-N diodes.

### 2.1. Simulation Analysis of Different Terminal Structures

**Figure 2:**
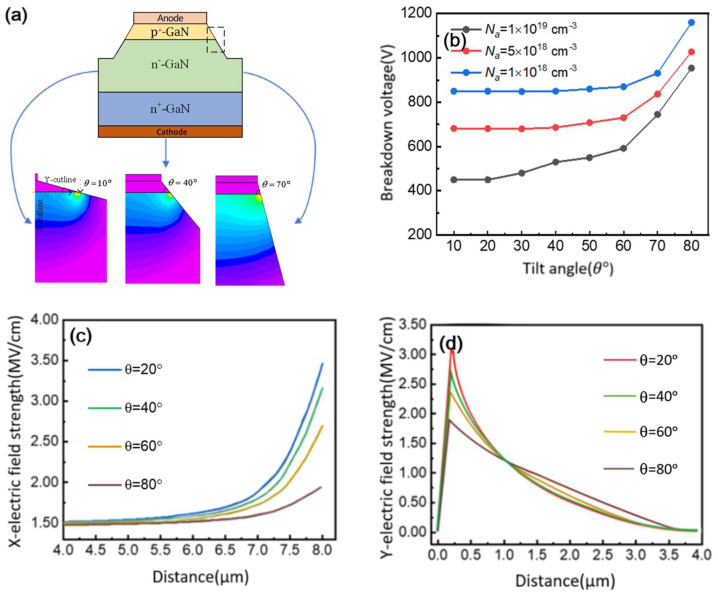
(**a**) The terminal structure at the edge of inclined mesa and the electric field distribution of P-i-N diodes with different tilt angles with a reverse bias of 350 V; (**b**) breakdown voltages of GaN P-i-N diodes with different tilt angles; (**c**) electric field distribution along X-tangent; (**d**) electric field distribution along Y-tangent.

The inclined mesa termination structure, compared to conventional vertically stepped etching termination designs, is widely employed in wide-bandgap semiconductor devices due to its fabrication advantages, as illustrated in Figure 2a. Our analysis reveals that smaller etching angles (*θ*) produce narrower depletion regions and create significant electric field crowding at the p-n junction edges, leading to premature breakdown, as depicted in Figure 2b. As *θ* increases, the depletion region extends further into the device interior, reducing field crowding and enhancing breakdown voltage. Optimal angles yield field distributions that closely approximate those of ideal parallel-plane junctions. Figure 2c demonstrates the impact of p^+^-GaN doping concentration on breakdown voltage in non-punch-through configurations, showing that while breakdown voltage generally decreases with smaller *θ*, heavily doped p^+^-GaN layers exhibit more rapid voltage reduction compared to lightly doped counterparts, which maintain higher voltages even at small angles.

The angle-dependent electric field evolution is presented in Figure 2d, where increasing *θ* from 20° to 80° reduces the horizontal field strength from 3.6 MV/cm to 1.8 MV/cm and the vertical field from 3.3 MV/cm to 1.7 MV/cm. These results clearly indicate that larger etching angles promote superior field uniformity and higher breakdown voltages. For devices with small angles, reducing the p^+^-GaN doping concentration can help maintain satisfactory breakdown performance. Furthermore, applying high-κ dielectric passivation layers on the mesa sidewalls provides additional benefits by extending the depletion region inward, smoothing the electric field distribution, and suppressing leakage currents caused by surface damage.

The one-dimensional field profiles presented in Figure 2d demonstrate that the Si_3_N_4_-passivated device maintains field magnitudes around 3.5 MV/cm at device edges, similar to conventional structures. However, the peak fields show substantial reduction, with the X-direction field decreasing from 3.5 to 2.5 MV/cm and the Y-direction field dropping from 3.5 to 2.0 MV/cm. This enhanced field uniformity, combined with the dielectric material’s excellent voltage withstand capability, allows the breakdown performance to approach that of ideal parallel-plane junctions.

### 2.2. Simulation Analysis of Field-Plate Mesa Termination Structure

**Figure 3:**
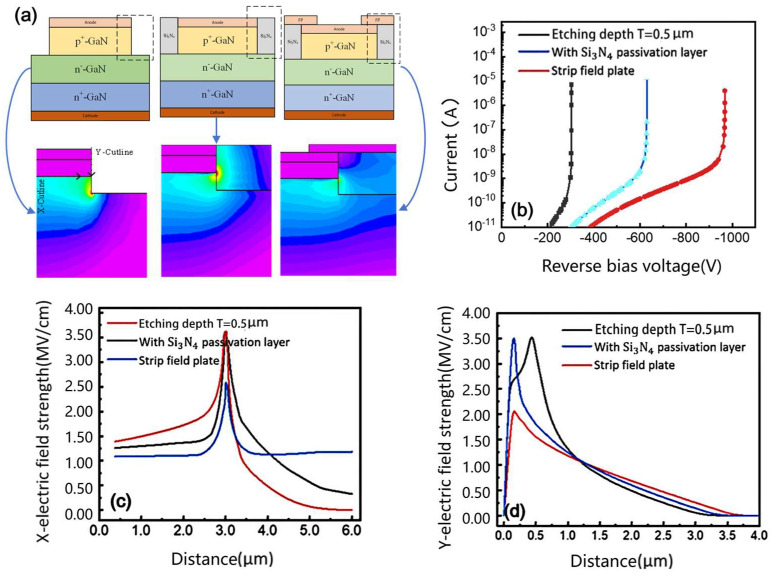
(**a**) Vertical mesa GaN P-i-N diode/with Si_3_N_4_ passivation layer/with field-plate structure and electric field distribution of GaN P-i-N diode with tilted mesa reverse bias of 300 V/structure with Si_3_N_4_ passivation layer/with field-plate structure; (**b**) breakdown voltage of GaN P-i-N diodes with three structures; (**c**) electric field distribution along X-tangent; (**d**) electric field distribution along Y-tangent.

Research has shown that preventing premature avalanche breakdown at device edges is crucial for improving breakdown voltage. The field-plate technique, a widely used edge termination method, enhances device performance by modifying the electric field distribution. When reverse bias is applied through the shared anode voltage source, electrons are repelled from the surface while the depletion region extends inward. This mechanism effectively reduces edge field crowding, thereby increasing the breakdown voltage. Figure 3a presents a comparative analysis of three vertical mesa configurations with 0.5 µm etching depth, alongside their corresponding electric field distributions. The results demonstrate that the Si_3_N_4_ passivation layer achieves superior field uniformity by relocating the peak electric field from the semiconductor edge to the dielectric interface. While localized field crowding persists at the p-n junction/dielectric boundary, the high critical breakdown strength of Si_3_N_4_ ensures robust operation by confining the maximum field within the more resilient dielectric material.

The field-plate structure further improves performance by extending the depletion region inward through the applied anode potential, resulting in smoother field distribution and significantly reduced edge field crowding. Breakdown voltage measurements in Figure 3b show that the Si_3_N_4_-passivated structure outperforms the simple etched mesa. This improvement stems from dielectric constant mismatch at the interface, which suppresses the electric field in the semiconductor adjacent to the high-κ Si_3_N_4_ layer. The one-dimensional field profiles in Figure 3c,d show that the Si_3_N_4_-passivated device maintains field magnitudes comparable to conventional structures at device edges, reaching approximately 3.5 MV/cm. However, it achieves a substantial reduction in peak fields, with the X-direction decreasing from 3.5 to 2.5 MV/cm and the Y-direction dropping from 3.5 to 2.0 MV/cm. This improvement in field uniformity, along with the dielectric’s superior voltage tolerance, allows the breakdown characteristics to approach those of ideal parallel-plane junctions.

**Figure 4:**
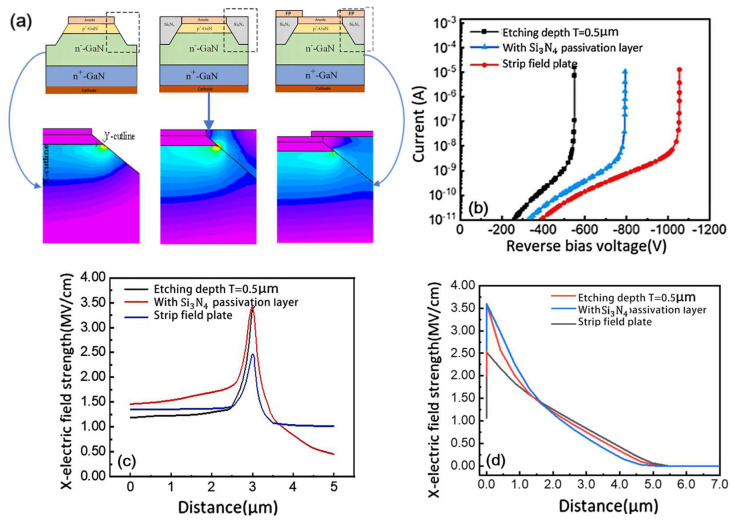
(**a**) Inclined mesa GaN P-i-N diode/with Si_3_N_4_ passivation layer/with field-plate structure and electric field distribution of GaN P-i-N diode with tilted mesa reverse bias of 300 V/structure with Si_3_N_4_ passivation layer/with field-plate structure; (**b**) breakdown voltage of GaN P-i-N diodes with three structures; (**c**) electric field distribution along X-tangent; (**d**) electric field distribution along Y-tangent.

Figure 4a presents both the simulated 40° inclined mesa structure and its corresponding electric field distribution under 300 V reverse bias, comparing three key configurations: the basic inclined mesa, Si_3_N_4_-passivated mesa, and field-plate implementation. The results clearly show that incorporating the Si_3_N_4_ passivation layer effectively transfers the peak electric field into the dielectric material. This modified configuration demonstrates significant improvements over the simple inclined mesa structure, achieving both deeper extension of the depletion region into the device and a more favorable gradual field distribution profile.

The field-plate structure further enhances performance by repelling surface electrons and extending the depletion region inward, thereby mitigating field crowding effects and improving breakdown voltage. Breakdown voltage measurements in Figure 4b reveal significant improvements: the Si_3_N_4_-passivated structure shows a 250 V increase, while the field-plate configuration achieves a 505 V enhancement over the basic inclined mesa. Figure 4c,d provide additional evidence that the Si_3_N_4_ passivation improves breakdown voltage by internalizing the peak electric field, while the field plate reduces field crowding at the p-n junction edge. The combined effect of field-plate and mesa structures substantially weakens edge field accumulation, yielding significantly higher breakdown voltages than the simple inclined mesa configuration.

## 3. Multi-Drift-Layer Structure Design of GaN-Based P-i-N Diode

### 3.1. Top P-GaN Region and Bottom N-GaN Region Design

For vertical GaN P-i-N diodes, the epitaxial structure typically incorporates p-type GaN layers comprising both heavily doped and moderately doped regions. In this design, we utilize a 10 nm thick p^+^-GaN layer with a doping concentration of 1 × 10^20^ cm^−3^ combined with a 500 nm thick p-GaN layer doped at 1.5 × 10^18^ cm^−3^. These doping concentrations are substantially higher than those in the drift region, which minimizes voltage drop and effectively creates a one-sided abrupt junction. The heavily doped p^+^-GaN layer mainly functions to enable optimal p-type ohmic contact formation.

Due to longstanding challenges in the GaN p-type doping technology, achieving both high doping concentrations and substantial thickness in p^+^-GaN layers remains difficult. Consequently, the simulated structure maintains this practical thin-layer configuration. The bottom n-GaN buffer layer, designed with 1 × 10^18^ cm^−3^ doping and 2 µm thickness, similarly exceeds the drift region doping while ensuring a good n-type ohmic contact formation.

### 3.2. Drift Region Design

In P-i-N power diodes under reverse bias, the depletion region expands within the lightly doped drift region. The device’s breakdown characteristics are determined by carefully designing the drift region thickness and doping concentration to achieve optimal trade-offs between breakdown voltage and on-resistance.

As reverse voltage increases, the depletion region extends through the drift region, with the peak electric field occurring at the n^-^drift and p^+^ interface. Breakdown occurs when *E_m_* exceeds GaN’s critical field strength. In non-punch-through designs, the electric field shows a triangular distribution, reaching zero at the n^+^-GaN substrate. The breakdown voltage corresponds to the integral of this field distribution.

Higher drift region doping concentrations prematurely terminate the depletion region before reaching the substrate, reducing breakdown voltage. Optimal design requires balancing doping and thickness to achieve complete depletion at the target voltage. Punch-through designs with lower effective doping create trapezoidal field distributions that provide higher breakdown voltages for the same thickness when *E_m_* equals *E_C_* at full depletion.

**Figure 5:**
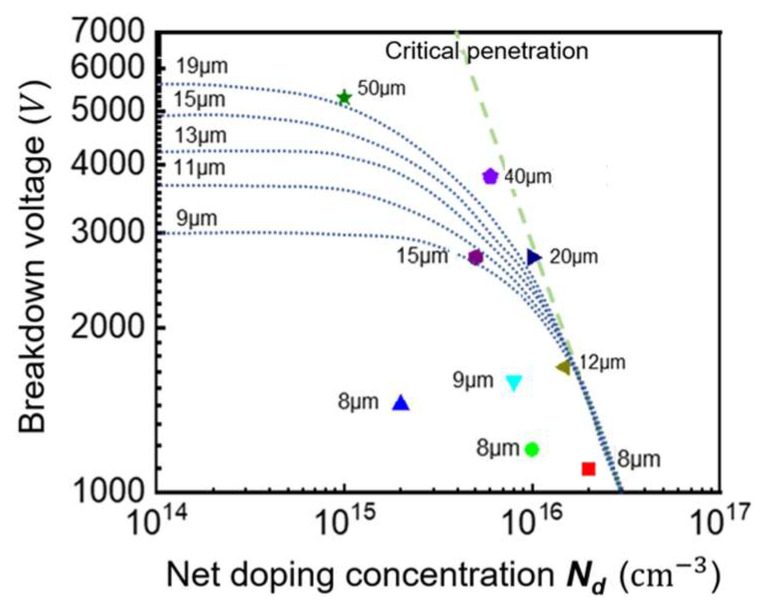
Relationship between breakdown voltage and doping concentration of GaN P-i-N diode at different drift-layer thicknesses [16,17,18,19,20,21,22,23,24].

Figure 5 illustrates how breakdown voltage varies with doping concentration for different drift-layer thicknesses. Three distinct regions are evident, i.e., near-punch-through, breakdown voltage increases sharply with decreasing doping; at lower doping, the increase becomes gradual; and at very low doping, the voltage saturates. While punch-through designs offer higher breakdown voltages, non-punch-through structures demonstrate superior switching performance with lower switching losses and reduced trailing current.

The design must therefore consider both static breakdown requirements and dynamic switching performance when selecting the drift region parameters. This involves the careful optimization of the doping profile and thickness to meet specific application requirements. When the device is close to the critical punch through, that is, at the inflection point of the curve in Figure 5, this is the best design structure of the theoretical device. Some vertical GaN P-i-N diodes reported so far are summarized and plotted in Figure 5 [16,17,18,19,20,21,22,23,24,25]. It can be found that most of the reported device structures are far from the theoretical optimal value.

**Figure 6:**
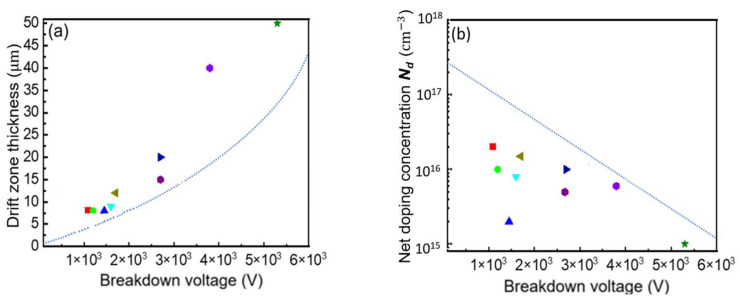
Drift parameters and breakdown voltage of GaN P-i-N diodes have been reported in part [16,17,18,19,20,21,22,23,24]. (**a**) Thickness and breakdown voltage; (**b**) doping concentration and breakdown voltage.

Figure 6 presents the required drift region thickness and effective doping concentration for GaN P-i-N diodes to achieve critical breakdown at various voltage levels. Experimental data reveal significant deviations from theoretical predictions: most devices exhibit drift layers that are over 50% thicker than theoretically optimal layers, while their effective doping concentrations often fall more than one order of magnitude below design values. These observations indicate a substantial gap between actual device parameters and theoretical optima, with practical implementations consistently featuring thicker and more lightly doped drift regions than calculations would suggest.

### 3.3. Device Structure

**Figure 7:**
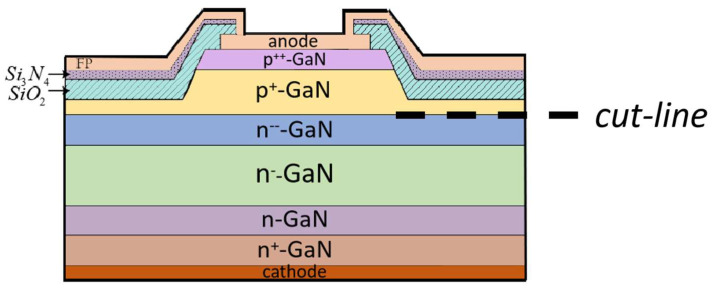
Schematic diagram of GaN P-i-N diode with triple-drift-layer and FP structure.

Based on the previous drift-layer analysis, this simulation investigates GaN P-i-N diodes with three configurations: single-drift-layer (SDL), double-drift-layer (DDL), and triple-drift-layer (TDL) structures. For the consistent comparison of doping concentration and length ratio effects, all designs maintain a total drift-layer thickness of 30 µm. Figure 7 illustrates the three-layer drift structure with field-plate termination. Compared to conventional designs, this structure features complete mesa coverage by field-plate-extended metal and a composite passivation layer using two dielectric materials [26,27]. The dielectric constant of Si_3_N_4_ is 7.5, and that of SiO_2_ is 3.9. This configuration effectively reduces reverse leakage current while enhancing breakdown voltage performance.

**Figure 8:**
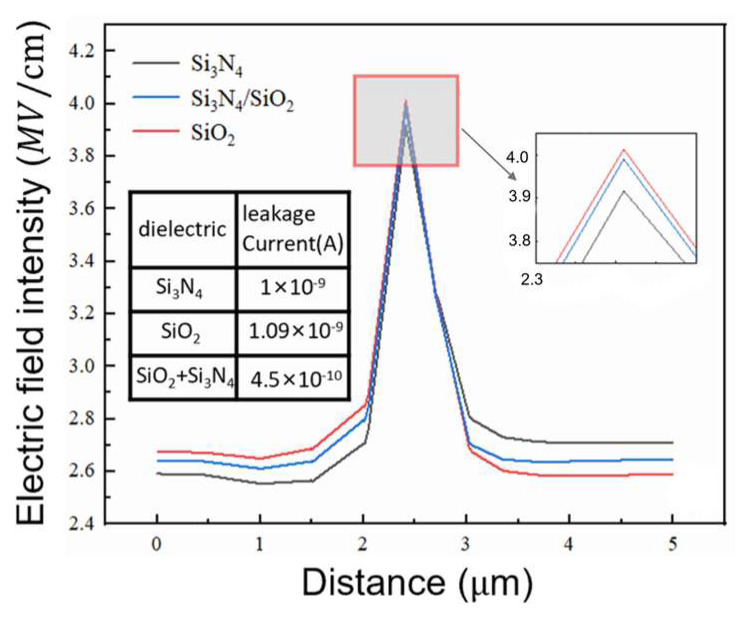
Comparison of electric field intensity distribution and leakage current in different dielectric layers.

The following Figure 8 shows the electric field intensity distribution along the cut-line of the device under the same reverse bias voltage. It can be observed that the peak surface breakdown electric field of the Si_3_N_4_ dielectric layer is lower compared to that of the oxide layer and the composite dielectric layer formed by the two. Due to the effect of the field plate, the Si_3_N_4_ layer, SiO_2_ layer, and composite dielectric layer all reduce the electric field intensity inside the depletion region and improve the uniformity of the electric field. Although the peak electric field of the composite field-plate layer is lower than that corresponding to the SiO_2_ layer, the composite field-plate leads to a more uniform surface electric field distribution.

**Figure 9:**
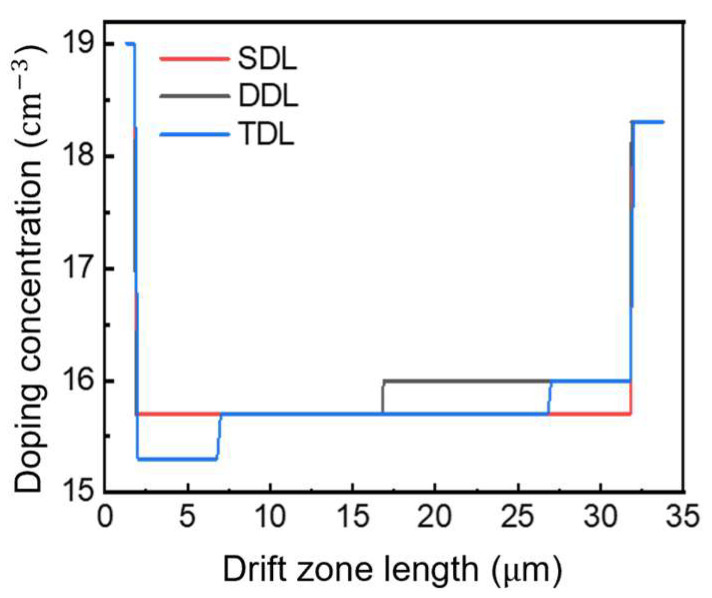
Doping concentration distribution curve of GaN P-i-N diode with three structures.

The surface leakage current values of the device under the same reverse bias voltage are further extracted and labeled in the above figure. It can be seen that the composite field plate reduces the surface leakage current more effectively compared to the Si_3_N_4_ and SiO_2_ dielectrics. Therefore, the use of a composite dielectric layer can combine the characteristics of both dielectrics, optimizing the electric field distribution and reducing leakage current to a certain extent. Figure 9 shows the ratio of doping concentration and drift region length of the three structures. Table 2 details the structural parameters of three different drift-layer devices.

## 4. Forward Conduction and Conductance Modulation Characteristics of GaN P-i-N Diode

**Figure 10:**
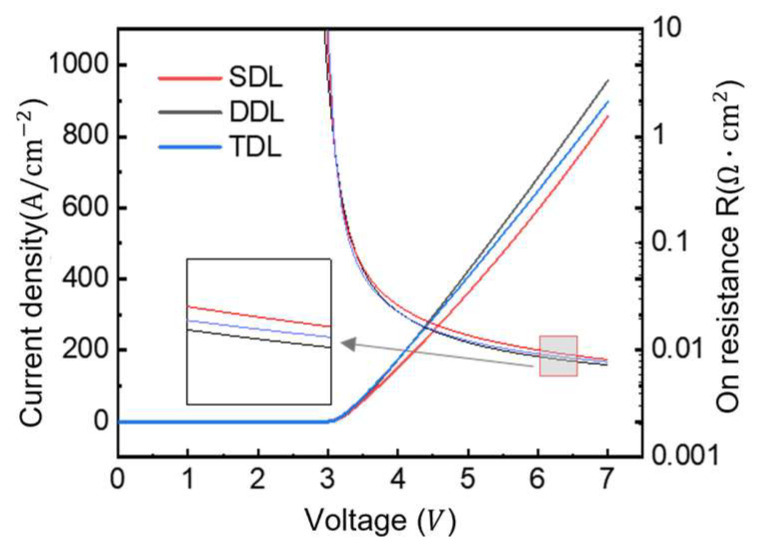
Forward characteristics of GaN P-i-N diodes with three structures.

Using the structural parameters described previously, we performed the TCAD simulations of GaN P-i-N diodes with three different drift-layer configurations. Figure 10 shows their forward conduction characteristics under applied bias. All three structures exhibit similar turn-on behavior, with a threshold voltage around 3 V and decreasing on-resistance at higher voltages due to conductivity modulation.

The DDL structure demonstrates slightly enhanced current characteristics relative to other configurations. This performance enhancement stems from its elevated doping concentration in the secondary layer, which effectively diminishes the on-resistance. Experimental validation through calculated on-resistance measurements shows the SDL structure at 8.17 mΩ·cm^2^, the DDL at 7.31 mΩ·cm^2^, and the TDL at 7.81 mΩ·cm^2^. These quantitative findings demonstrate direct correlation with the doping profiles presented in Figure 9, clearly illustrating that enhanced drift region doping consistently yields reduced on-resistance values.

**Figure 11:**
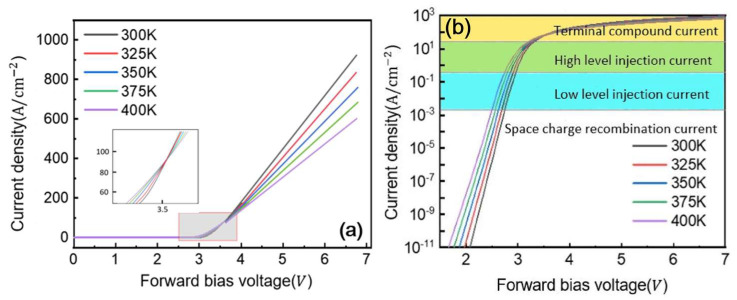
Forward characteristics of TDL with different temperature. (**a**) Forward characteristic of linear scale; (**b**) forward characteristic of logarithmic scale.

The proposed structure effectively suppresses electric field crowding at junction edges through field-plate optimization while reducing on-resistance via a multi-layer n-type drift region with graded doping concentrations. Although increasing the overall doping concentration in the drift region improves forward characteristics by lowering on-resistance, this enhancement comes at the expense of reduced breakdown voltage, necessitating careful design trade-offs to achieve optimal device performance. Temperature-dependent simulations reveal important effects on forward conduction characteristics, as shown in Figure 11 for three-layer drift GaN P-i-N diodes. As temperature increases from 300 K to 400 K, we observe a slight but consistent decrease in turn-on voltage along with a distinct crossover point in the current–voltage characteristics. The temperature dependence exhibits two distinct regimes: at low injection currents, forward current increases with temperature, while at high currents (exceeding 80 A/cm^2^), the behavior reverses due to competing mechanisms. While carrier lifetime enhancement with temperature reduces voltage drop, mobility degradation dominates at higher current densities, leading to increased dynamic on-resistance. This transition occurs when the drift region carrier density becomes insufficient to maintain effective conductivity modulation, ultimately resulting in elevated conduction losses at elevated temperatures, as clearly demonstrated in Figure 11a,b; this figure, presented in a logarithmic scale, reveals four distinct conduction mechanisms in the P-i-N diode, characterized by different slopes representing space charge recombination, low injection, high injection, and junction recombination regimes. A significant increase in forward voltage drop occurs above 80 A/cm^2^ due to junction-resistance effects, reaching 3.7 V at 100 A/cm^2^ and 300 K.

## 5. Reverse Breakdown Characteristics of GaN-Based P-i-N Diodes

**Figure 12:**
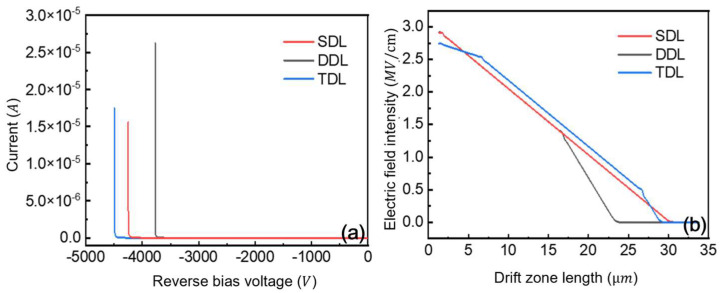
(**a**) Reverse characteristics of three GaN P-i-N diodes with three structures. (**b**) Electric field distribution during avalanche breakdown with three structures.

This study models and simulates three GaN P-i-N diode configurations with different drift-layer structures. Figure 12a presents their reverse breakdown characteristics, demonstrating excellent performance across all designs. The TDL structure achieves the highest breakdown voltage of 4494 V while even the DDL configuration maintains a substantial 3772 V breakdown capability. These results stem from two key design features: a 30 µm total drift region with low overall doping concentration, and an optimized field-plate termination incorporating a composite dielectric passivation layer. The field-plate structure effectively modulates potential distribution under reverse bias, mitigating field crowding and reducing leakage current while enhancing breakdown voltage performance. Figure 12b presents the one-dimensional electric field distributions at avalanche breakdown for the three device structures. All configurations employ non-punch-through designs, with the electric field slope varying according to drift region doping concentrations. Lower doping concentrations yield gentler field gradients, while the breakdown voltage corresponds to the area under each field distribution curve.

The DDL structure suffers from premature depletion region termination caused by higher doping concentrations in its secondary layer. This leads to the incomplete utilization of the designed drift region length and ultimately results in lower breakdown voltages when compared to alternative designs. In contrast, the TDL structure demonstrates enhanced breakdown performance through two distinct mechanisms. First, its reduced doping concentration in the initial layer enables broader electric field distribution. Second, the elevated doping level in the tertiary layer preserves non-punch-through characteristics while simultaneously maintaining favorable forward conduction properties. These results demonstrate that optimal breakdown voltage requires careful balance between drift region doping concentration and length. While terminal structures help maintain breakdown performance near ideal levels, fundamental improvement ultimately depends on either reducing doping concentrations or increasing drift region dimensions.

**Figure 13:**
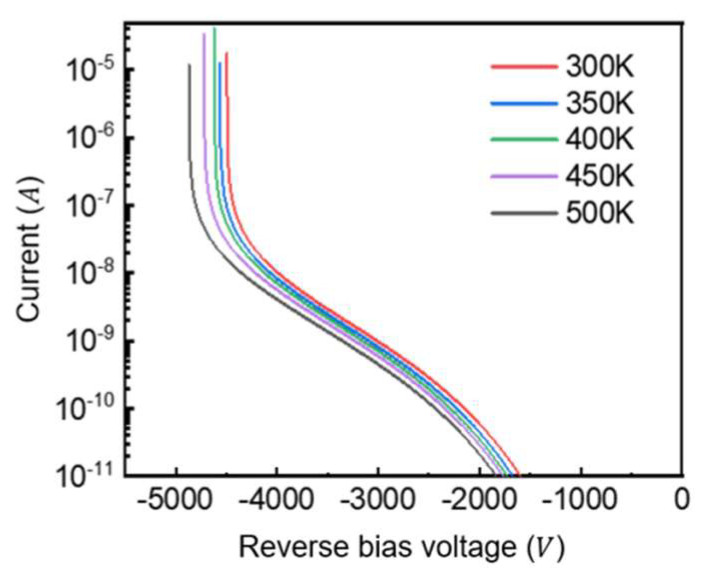
Reverse breakdown characteristics of TDL at different temperatures.

In practical applications, the power P-i-N diode is usually required to work in the temperature range of about 250 K to more than 400 K. The temperature dependence of breakdown voltage originates from the critical electric field strength, which is governed by impact ionization rates. Since these ionization rates decrease with rising temperature, we investigated this effect in our three-layer drift GaN P-i-N diode with complex field-plate structure. As shown in Figure 13, the breakdown voltage increases from 4494 V at 300 K to 4870 V at 500 K, demonstrating the positive temperature coefficient behavior characteristic of avalanche breakdown mechanisms. This trend confirms the device maintains stable avalanche breakdown characteristics across the operational temperature range.

## 6. GaN-Based P-i-N Diode Switching Characteristics

The unidirectional conductivity of P-i-N diodes makes them ideal for switching applications, where they function as near-short circuits under forward bias and open circuits in reverse bias. These properties are particularly valuable in switch-mode power supplies, though practical implementations must minimize switching time and losses.

### 6.1. Forward Recovery Characteristics

**Figure 14:**
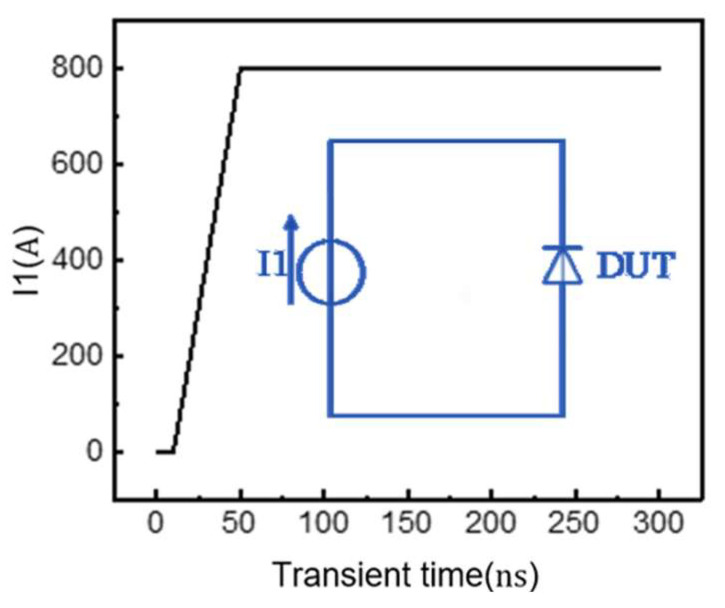
Forward recovery simulation circuit and current source waveform.

We investigated forward recovery behavior through device–circuit co-simulation. Figure 14 illustrates the simplified test circuit, where current source I1 provides a controlled d*i*/d*t* ramp to characterize the transient turn-on behavior of the device under test. This approach effectively simulates the instantaneous conduction characteristics of the P-i-N diode during switching events.

**Figure 15:**
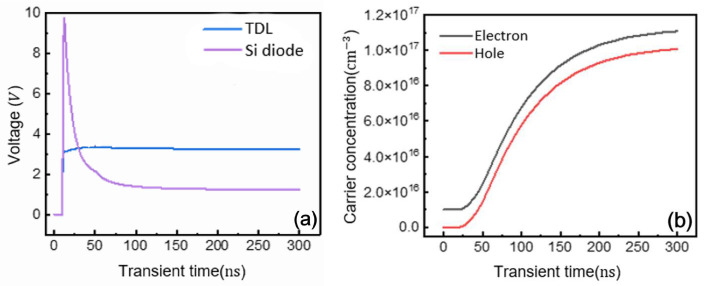
Forward recovery process voltage waveform and carrier concentration waveform. (**a**) Voltage waveform; (**b**) carrier concentration waveform.

Figure 15a compares the forward recovery characteristics between GaN and Si P-i-N diodes with various drift-layer configurations. Under an 800 A current step, Si diodes exhibit approximately 10 V voltage overshoot, whereas GaN devices show minimal overshoot. This difference originates from their distinct drift-layer geometries—the thick drift region in Si diodes requires substantial charge injection during turn-on transients, while GaN diodes with their thin drift layers achieve conduction with negligible overshoot. Consequently, GaN P-i-N diodes demonstrate significantly lower energy losses during forward recovery, with all triple-drift-layer designs exhibiting similar performance characteristics. Figure 15b illustrates carrier dynamics during forward recovery, showing how electron and hole injection progressively enhances conductivity modulation. The carrier concentration stabilizes once forward recovery completes, indicating established conductance modulation.

For many years, P-i-N diode turn-on characteristics received little research attention. However, in practical circuits, the voltage overshoot during turn-on can potentially damage reverse-parallel connected devices. Furthermore, the turn-on energy loss typically represents only a few percent of either turn-off or conduction losses, making it negligible in most applications.

### 6.2. Reverse Recovery Characteristics

**Figure 16:**
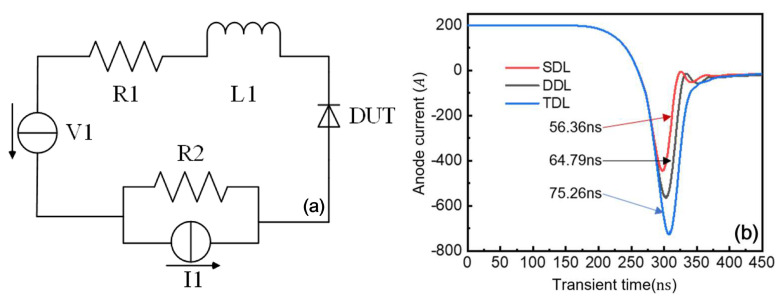
(**a**) Reverse recovery simulation circuit; (**b**) reverse recovery current curves of three GaN P-i-N diodes with different structures.

By using the device circuit hybrid simulation module of the TCAD tool, the double-pulse simulation is carried out to study the reverse recovery characteristics in a specific circuit. Figure 16a shows a simplified diagram of P-i-N diode reverse recovery simulation circuit, where I1 and V1 are voltage source and current source, respectively, and the rated voltage and current are 400 V and 200 a, respectively; R1 resistance is 1 mΩ; L1 is inductance, and the size is 2 µH.

The reverse recovery characteristics were analyzed using a two-stage device–circuit co-simulation approach. First, steady-state operation was established, followed by the transient analysis of the reverse recovery process. This was implemented through the exponential reduction in output resistance R2 from 1 mΩ to 1 µΩ, effectively creating a controlled short-circuit condition for the parallel-current source I1. The current decay dynamics during reverse recovery are governed by the circuit’s voltage source and inductance parameters. During the transition from forward to reverse bias, distinct negative current and voltage peaks emerge, influenced by both the device architecture and circuit conditions, particularly the current decay rate. As the anode–cathode polarity reverses, the current exhibits a transient reverse flow while evacuating stored charge from the forward-conduction phase. The reverse recovery time is conventionally measured from current zero-crossing to the point where it decays to 10% of its peak reverse value.

Figure 16b compares the reverse recovery characteristics of GaN P-i-N diodes with different drift-layer configurations. The TDL structure demonstrates more pronounced recovery behavior with a 727 A peak reverse current and 75.26 ns recovery duration, while the SDL variant shows improved performance with 564 A peak current and 56.36 ns recovery time. These differences primarily stem from variations in charge storage capacity and extraction dynamics inherent to each structural design.

**Figure 17:**
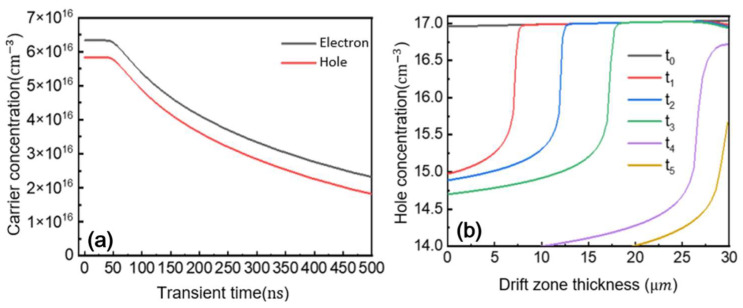
Change in carrier concentration in reverse recovery process. (**a**) Change in carrier concentration; (**b**) change in hole concentration in drift region.

Figure 17a shows the transient carrier concentration at a fixed point in the drift region during reverse recovery. The decreasing concentration demonstrates the need to remove free carriers that were injected during forward conduction. Figure 17b presents the minority hole distribution in the drift region at different stages of reverse recovery. Initially, the entire drift region contains a high concentration of minority holes. As reverse recovery progresses, holes are first extracted from the top portion of the drift region, allowing the depletion region to gradually expand downward to support the applied reverse voltage.

**Figure 18:**
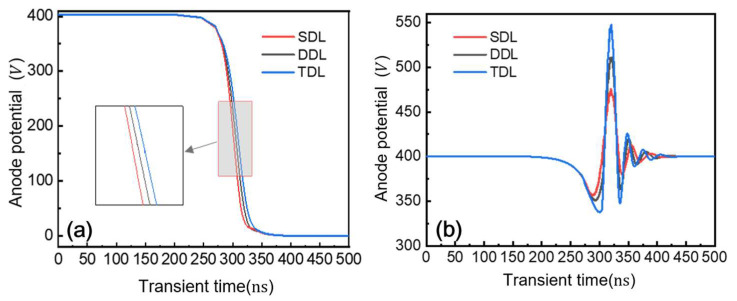
Reverse recovery process potential change. (**a**) Anode potential curve; (**b**) cathode potential curve.

During reverse recovery, the inductor’s electromotive force adds to the diode voltage, causing the peak reverse voltage to exceed the 400 V source rating by up to 400 V. Figure 18 shows the potential variations at the diode’s anode and cathode during this process. In Figure 18a, the anode potential drops from the 400 V source rating to 0 V when the current source is short-circuited by reducing R2. Figure 18b reveals the cathode potential peaks for three structures: the TDL reaches approximately 550 V, while the DDL and SDL peak at 510 V and 470 V, respectively. All three structures exhibit terminal voltages significantly exceeding the 400 V source rating. The simultaneous occurrence of current and voltage peaks during reverse recovery generates high transient power, making the diode’s power loss non-negligible. The reverse recovery characteristics of P-i-N power diodes are typically evaluated under specific temperature conditions, forward current levels, and current fall rates (di/dt). This section analyzes how these parameters affect the reverse recovery process in a triple-drift-layer field-plate P-i-N diode.

High temperatures generally degrade reverse recovery performance. At fixed forward current and reverse bias, elevated temperature cannot generate additional carriers from fully ionized impurities. While majority carrier concentration remains stable, minority carriers increase more rapidly, requiring longer reverse recovery times to remove them.

**Figure 19:**
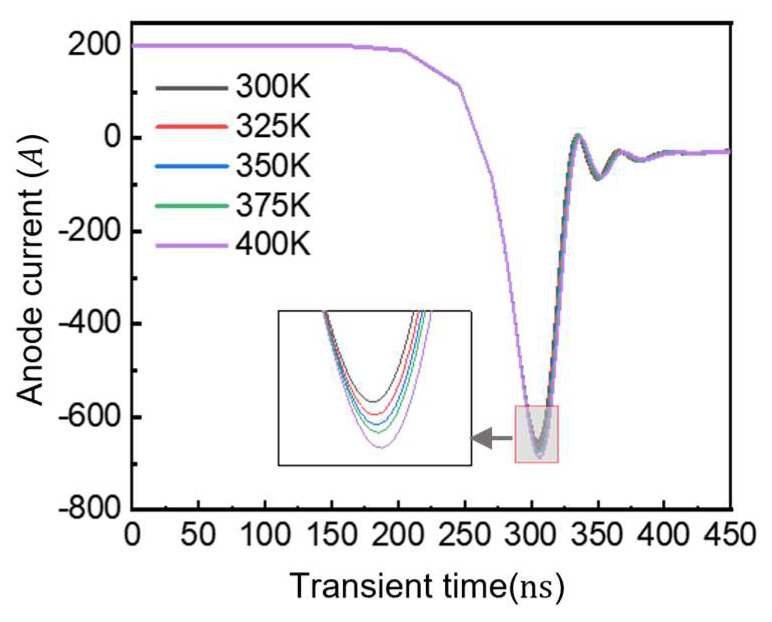
Reverse recovery characteristics at different temperatures.

Figure 19 presents the temperature-dependent reverse recovery characteristics of GaN diodes. Remarkably, when temperature increases from 300 K to 400 K, GaN P-i-N diodes show minimal changes in reverse recovery behavior, with only a slight increase in reverse current peak. This thermal stability stems from GaN’s wide bandgap, demonstrating excellent high-temperature turn-off capability.

**Figure 20:**
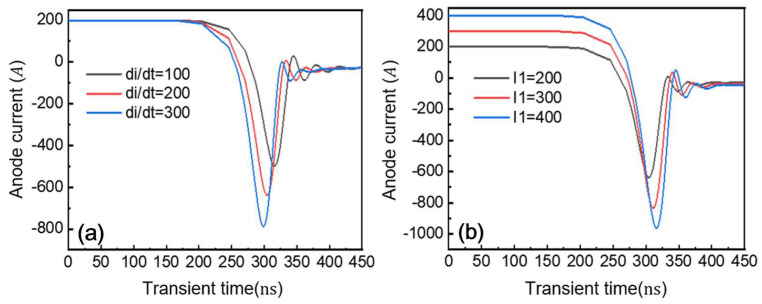
Reverse recovery process anode current waveform. (**a**) Variation in current drop rate; (**b**) variation in forward current.

Figure 20a illustrates the impact of current fall rate (di/dt) on reverse recovery characteristics. The reverse peak current exhibits a direct correlation with di/dt, reaching 496 A, 638 A, and 786 A at di/dt values of 100, 200, and 300 kA/ (cm^2^·μs), respectively. Lower di/dt results in a more gradual current transition during reverse recovery, producing both smaller peak currents and lower residual charge. The reverse recovery peak current, being proportional to the turn-off di/dt, critically impacts system performance. Higher peak currents not only increase diode power dissipation but also elevate primary circuit current, consequently raising switching losses. This relationship ultimately limits the maximum usable switching frequency in GaN P-i-N diode power electronic systems. Figure 20b demonstrates the reverse recovery characteristics under varying forward current conditions. Simulation results show that both the reverse recovery peak current and duration increase with higher initial forward currents. When the forward current rises from 200 A to 300 A and 400 A, the corresponding reverse peak currents measure 638 A, 832 A, and 963 A, respectively. This correlation occurs because larger forward currents inject more charge into the drift region during steady-state operation, requiring longer extraction times when reverse bias is applied.

To reduce the recovery time of the triple-drift-layer device, we leverage the dependence of PiN diode current fall rate and forward current magnitude on external circuits. This suggests that increasing the current fall rate and optimizing the forward current magnitude can effectively reduce the reverse recovery time. A higher current fall rate accelerates the extraction of stored charges in the intrinsic region, while appropriate adjustment of the forward current magnitude balances charge storage and extraction, thereby shortening the reverse recovery time and reducing power losses simultaneously.

## 7. Comparison of Simulation Results

The breakdown voltage and on-resistance of GaN P-i-N diode with triple-drift-layer field-plate terminal structure designed in this paper are compared with those in other references, as shown in Table 3.

The breakdown voltage and on-resistance mainly depend on the structural parameters of the drift region, that is, high breakdown voltage requires a long drift region length and a low doping concentration, while low on-resistance requires a high doping concentration. As can be seen from Table 3, the triple-drift-layer GaN P-i-N diode designed in this paper has a total length of 30 µm in the drift region, achieving a breakdown voltage of 4494 V, which is higher than that in Ref. [22], but lower than that in Ref. [24]. Because the overall doping level is lower than that in Refs. [22,25], the on-resistance is also higher, but it is still suitable for high voltage and high current applications.

## 8. Conclusions

This work presents critical design insights for vertical GaN P-i-N diodes through the systematic exploration of advanced edge termination and multi-drift-layer architectures. Key findings reveal that integrating field-plate termination with optimized mesa etching effectively mitigates electric field crowding, enabling breakdown voltage enhancement without compromising forward conduction. The multi-drift-layer design, featuring graded doping profiles, achieves a strategic balance between breakdown capability and on-resistance by tailoring electric field distribution and conductivity modulation. Practical design guidelines derived from this study emphasize the use of composite field-plate structures to redistribute peak fields into high-κ dielectrics for improved edge termination. The optimization of drift-layer doping gradients is also critical to balance high-voltage blocking and low on-resistance. Additionally, the thermal stability benefits of GaN materials enable consistent avalanche breakdown behavior across operational temperatures. These structural recommendations address core trade-offs in GaN power device design, providing a robust framework for developing high-performance devices with reliable switching characteristics and scalable manufacturing viability.

## Figures and Tables

**Table 1:** Material and model parameters for GaN.

Material/Model	Property	Value
GaN	Bandgap (eV)	3.43
	Electron affinity (eV)	4.1
	Dielectric constant	8.9
	Effective Conduction Band Density of states (cm^−3^)	2.24 × 10^18^
	Effective Valence Band Density of states (cm^−3^)	2.51 × 10^19^
Impact Ionization	*a_n_*_1/2_ (cm^−1^)	2.52 × 10^8^
	*b_n_*_1/2_ (V/cm)	3.41 × 10^7^
	*a_p_*_1/2_ (cm^−1^)	5.37 × 10^6^
	*b_p_*_1/2_ (V/cm)	1.96 × 10^7^
Incomplete Ionization	Donor Activation Energy (Δ*E_D_*) (meV)	17
	*α*_n_ (eV.cm)	3.4 × 10^−9^
	Acceptor Activation Energy (Δ*E_A_*) (meV)	240
	*α_p_* (eV.cm)	1.15 × 10^−9^
SRH Recombination	Electron Lifetime (*τ*_n_)	1.2 × 10^−8^
	Hole Lifetime (*τ_p_*)	1.2 × 10^−8^
Auger Recombination	Electron Coefficient (cm^6^∙s)	2.8 × 10^−31^
	Hole Coefficient (cm^6^∙s)	9.9 × 10^−32^

**Table 2:** Structural parameters of three GaN P-i-N diodes with different drift layer configurations. (DCR stands for Doping Concentration Range.)

Structural	DCR	SDL	DDL	TDL
p^++^-GaN	>1 × 10^19^ cm^−3^	1 × 10^20^ cm^−3^/10 nm	1 × 10^20^ cm^−3^/10 nm	1 × 10^20^ cm^−3^/10 nm
P^+^-GaN	1 × 10^18^~1 × 10^19^ cm^−3^	2 × 10^18^ cm^−3^/500 nm	2 × 10^18^ cm^−3^/500 nm	2 × 10^18^ cm^−3^/500 nm
n^−−^-GaN	<5 × 10^15^ cm^−3^	—	—	2 × 10^15^ cm^−3^/5 µm
n^−^-GaN	5 × 10^15^~ 1 × 10^16^ cm^−3^	5 × 10^15^ cm^−3^/30 µm	5 × 10^15^ cm^−3^/15 µm	5 × 10^15^ cm^−3^/20 µm
n-GaN	1 × 10^16^~ 1 × 10^18^ cm^−3^	—	1 × 10^16^ cm^−3^/15 µm	1 × 10^16^ cm^−3^/5 µm
n^+^-GaN	1 × 10^18^~1 × 10^19^ cm^−3^	2 × 10^18^ cm^−3^/2 µm	2 × 10^18^ cm^−3^/2 µm	2 × 10^18^ cm^−3^/2 µm

**Table 3:** Comparison of performance parameters of different GaN P-i-N diodes.

Structural and Performance Parameters	This Work	Ref. [22]	Ref. [24]	Ref. [25]
Terminal technology type	Field plate	Ion implantation	Junction terminal extension	Field plate
Drift region length (µm)	5/20/5	8	50	6/11/15
Doping concentration in drift region (cm^−3^)	2 × 10^15^/5 × 10^15^ /1 × 10^16^	1 × 10^16^	<1 × 10^15^	1 × 10^15^/3 × 10^15^ /1.2 × 10^16^
Breakdown voltage *V_B_* (V)	4494	1200	6400	3500
On-resistance *R_on_* (mΩ·cm^2^)	7.81	0.11	10.2	0.95
Substrate type	Bulk GaN	Bulk GaN	Bulk GaN	Bulk GaN
Fabrication technology	MOCVD	MOCVD	Solid-assisted separation	Hydride vapor phase epitaxy

## Data Availability

The datasets presented in this article are not readily available because the data are part of an ongoing study. Requests to access the datasets should be directed to the corresponding authors.
